# Anxiety towards Statistics and Its Relationship with Students’ Attitudes and Learning Approach

**DOI:** 10.3390/bs11030032

**Published:** 2021-03-10

**Authors:** Ángel Peiró-Signes, Oscar Trull, Marival Segarra-Oña, J. Carlos García-Díaz

**Affiliations:** 1 Management Department, Universitat Politècnica de València, 46022 Valencia, Spain; maseo@omp.upv.es; 2 Department of Applied Statistics and Operational Research and Quality, Universitat Politècnica de València, 46022 Valencia, Spain; otrull@eio.upv.es (O.T.); juagardi@eio.upv.es (J.C.G.-D.)

**Keywords:** statistics, attitudes, anxiety, deep learning, surface learning, fsQCA

## Abstract

Many university students have difficulties when facing statistics related tasks, leading to an increase in their levels of anxiety and poor performance. Researchers have identified negative attitudes towards statistics, which have been shaped through students’ secondary education experience, as a major driver for their failure. In this study we want to uncover the causal recipes of attitudes leading to high and low levels of anxiety in secondary education students, and the role that the learning approach plays in these relationships. We used fuzzy sets comparative qualitative analysis (fsQCA) in a sample of 325 students surveyed on the multifactorial scale of attitudes toward statistics (MSATS) and the revised two factor study process questionnaire (R-SPQ-2F). The results indicate that, respectively, a high or a low level of self-confidence is the most important and a sufficient condition by itself for achieving a low or a high level of anxiety, while the learning approaches and other attitudes are only present in other causal combinations that represent a small number of cases.

## 1. Introduction

Educators in higher education courses in the statistics area are concerned about their students’ learning. The search for effective learning and better performance has driven many studies in the past [1,2]. Research has found that attitudes are a crucial aspect in achieving both goals [3]. In fact, they are closely related to the assimilation and the capability to use content, and, therefore, to performance [4]. Good attitudes create a positive [5] and motivational [4] learning environment, while negative attitudes act as the major barrier to effective learning [6].

Attitudes are reactions to a situation and are made to manifest through emotions, conceptions or behavioral patterns [7,8]. These emotional responses, either positive or negative, are developed and automatized over time [3] and are composed of non-observable distinctive dimensions [7,8]. Researchers have identified and measured various attitudes towards statistics [5,9,10,11,12,13], including self-confidence [14] in the intellectual knowledge and skills for the subject, belief in the usefulness [14] of the subject in the future and expectations about their performance in the subject [15] or its difficulty.

Attitudes have been also highlighted as influencers of students’ learning [16] and performance [14,15,17,18] in statistics, and therefore need a lot of attention. Among the different attitudes towards statistics, anxiety has been uncovered as an important driver of poor academic performance in statistics [3,8,19,20,21,22,23,24,25,26]. Anxiety manifests itself as an emotion of apprehension, nervousness or concern when facing a situation [27,28]. The negative connotations of anxiety on performance have been widely studied in relation to statistics. The results of the significance of this relationship found in previous studies are contradictory [22]. These contradictory results seem to be affected by the different variables considered in the studies, by how the relationships between these variables are conceived [8,29] (i.e., as direct or mediated relationships) and by the context of the study. However, it is generally accepted that negative attitudes increase the level of anxiety and produce lower achievement [30].

The level of students’ anxiety increases with low values of self-perception and self- confidence [27], which are considered a big threat to their performance [31]. Self-perception and self-confidence are developed through past knowledge [6] and experience [22] in an area, and determine the student’s expectations [12]. The expectations and the value given by a student to a task affect his or her motivation [12], which has also been linked to students’ performance [9,32]. Consequently, attitudes, anxiety and performance are highly interrelated and have regularly been related to the student’s previous training in statistics or mathematics.

Although some authors have considered other characteristics of the students in their research, such as age [19,27,33] or gender [33,34,35], the results have been inconclusive. Furthermore, student learning is context-dependent [36]. For this reason, some authors have also considered in their studies, such as the teaching methodology [16,37,38,39,40,41] or the learning approach [40,42,43]. As regards the teaching methods, there has been a move towards methodologies in which students play a central role. There has been a switch to a more inductive way of learning, through “hands-on”, experiential activities [44,45,46,47,48] or other alternative methods [49], in a search for a change mainly in students’ motivation and enjoyment and, with activities close to real-life situations, an increase in the perceived usefulness, ultimately improving beliefs and the understanding of statistics [44,45,46,47,48,49,50], reducing anxiety and improving performance [37,38,51]. With regard to students’ strategies, students are typically classified as surface or deep learners. Surface learners tend to focus on the important points and on reproducing them, while deep learners go further, exploring, understanding, engaging and thinking critically. The learning approach is affected by many factors, including teaching and assessment methods, the perception of the task demands, and students’ motivations and values. The approach to a particular task also depends on the activities proposed (teaching context) and the students’ perceptions and characteristics. Students’ approaches to learning have also been treated as a matter of interest in the area of this study. Mondéjar et al. [40] reported higher levels of anxiety in surface versus deep learners, while Walsh and Ugumba-Agwunobi [52] found these higher levels in students with higher expectations. On the contrary, Daley and Onwuegbuzie [43] found lower levels of anxiety when students had a higher degree of mathematical and logical thinking.

Statistics and statistical related concepts are present in the syllabus of many undergraduate courses [16]. Improving students’ performance and attitudes towards statistics is not an easy task. In addition to the specific didactic problems caused by the heterogeneity in students’ mathematical backgrounds [53], educators have to deal with students’ attitudes towards the subject, which have been configured over time based on their previous experiences with statistics and mathematics in secondary education.

Despite the numerous studies about the attitudes towards statistics, the complex relationships between these attitudes suggest that more research needs to be conducted [54]. There is a need, first, to avoid the methodological issues that arise from the actual studies based on regression models arise (i.e., dealing with asymmetrical relationships between variables or the interpretation of multiple variables interactions), and, second, to include the learning approach, which is not considered in depth in these studies, especially in secondary education. A better knowledge of the relationships between attitudes towards statistics in secondary education, their interactions between these attitudes, and their relationship to the students’ learning strategies can help to provide an understanding of the problem and to allow teaching and learning activities to be planned in a way that is directed towards the creation of positive attitudes [55,56,57], a reduction in anxiety levels and an improvement in academic performance.

Previous closely related studies found in a limited sample some attitudes to be relevant in the causal combinations that lead to positive and negative levels of anxiety [29]. This study includes a broader sample and explores the role of the learning strategy in the relationship between students’ attitudes towards statistics and students’ anxiety. As indicated by Biggs et al. [58], in an ideal system we would expect students to have a deep approach to learning, which involves them engaging in the highest levels of learning. Anxiety is an emotion of concern when faced with a situation, whereas a deep or surface strategy reveals the way the student engages with the situation. We then expect that the way in which a student faces a situation to have a significant impact on their emotional state of concern, and therefore that learning strategy should be present in the causal combinations leading to low or high levels of anxiety.

The paper is organized as follows: Section 2 conducts a review of the measure instruments and fuzzy sets comparative qualitative analysis (fsQCA) methodology and an explanation of the sample used in the study; Section 3 presents the results and their analysis; and finally, in Section 4, we discuss the results and we summarize the conclusions and future research.

## 2. Materials and Methods

In this study we evaluated the role of the students’ approaches to learning on their anxiety towards statistics in secondary education. Statistics is included in mathematics courses from the 7th grade in the Spanish educational system. As in the first courses its presence is residual, we focused on those students who had minimum exposure that had allowed them to develop attitudes towards statistics. We surveyed students undertaking the three courses taken prior to the first year at the university that have mathematics as a mandatory subject. Our study comprises 10th graders (last year in compulsory secondary education) and 11th and 12th graders students (Baccalaureate courses) on science and social sciences courses from three different secondary schools (47.4%, 23.7% and 28.9% of the sample, respectively). These schools are located in three different suburban areas very close to Valencia in Spain. The questionnaires were passed by each math teacher in between October and December 2020 in their regular class sessions to assure a high response rate. After disregarding a few cases, we ended up with a sample of 325 students, 180 responses were from 10th graders (55.38%), 110 from 11th graders (33.85%) and the remaining 35 (10.77%) from 12th graders. Of the respondents 50.2% were male and 55.52% classified themselves as science students versus a 44.48% that did it as letters students. Students in the survey reported an average grade in mathematics of 6.86 over 10 (Std. Dev. = 1.57) and an overall average grade of 7.19 over 10 (Std. Dev. = 1.18).

In previous studies, researchers have evaluated anxiety together with other attitudes towards statistics. Among the usual instruments used to measure these attitudes we could find the statistics attitude survey (SAS) [10], the attitudes toward statistics scale (ATS) [11] and the survey of attitudes toward statistics scale (SATS) [12]. In Spain, some authors have developed similar scales based on these surveys, such those by Auzmendi [9], Estrada [5] and Bayot-Mestre et al. [13]. Although there are some differences in the names of the dimensions and the items used to measure these dimensions, most authors agree on three basic dimensions [9,34]; a dimension related to the feelings created by statistics (affective), a dimension related to the thoughts, conceptions and beliefs linked to statistics (cognitive) and a dimension linked to the behavior, either actional or intentional, in relation to the statistics (behavioral).

We decided to use Auzmendi’s multifactorial scale of attitudes toward statistics (MSATS) [9] because it was originally developed in Spanish and it is the most extensively used [59] and validated [9,59] in this language. The questionnaire (see Appendix A) evaluates five dimensions of attitudes towards statistics: self-confidence, usefulness, motivation, enjoyment and anxiety. They represent, respectively, the confidence that the student has when dealing with statistics, the value that knowledge of statistics represents for the student, the student’s driving force towards the study of statistics, the satisfaction and fun felt when performing statistical work and the concern or stress exhibited by the student when dealing with statistics. The five dimensions are measured with 5-point Likert scales, with 25 items and they are valued from 1 (“strongly disagree”) to 5 (“strongly agree”). As a result of the meaning direction of the questions related to anxiety and motivation, we reverse coded the item values related to these two dimensions to display positive attitudes as the number increases. Accordingly, high values represent high motivation or low anxiety.

Additionally, to evaluate the students’ learning strategy, we used Biggs et al.’s [58] revised two factor study process questionnaire (R-SPQ-2F). The R-SPQ-2F comprises 20 questions (see Appendix A) and also uses a 5-point Likert scale ranging from 5 (“always true of me”) to 1 (“only rarely true of me”). The survey additive scale results in two scores, one for deep and another for surface learning. We considered both learning approaches as causal conditions of the desired outcome in our analysis. A higher score means a higher approach to deep or surface learning, respectively.

**Figure 1:**
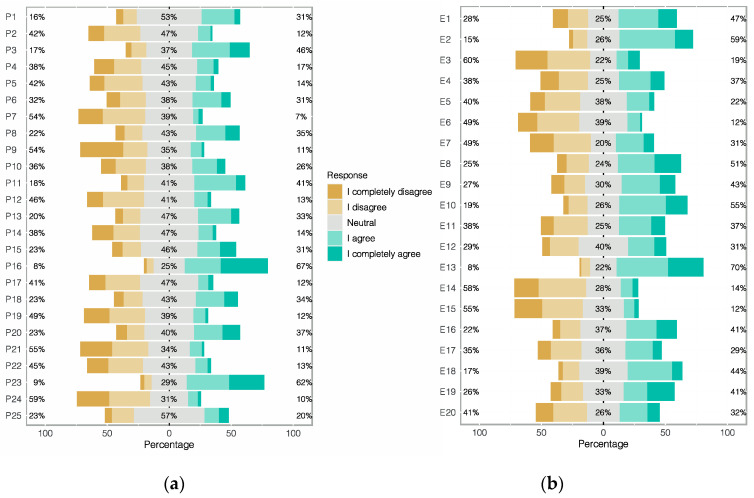
Distribution of students’ responses. (**a**) MSATS and (**b**) R-SPQ-2F.

Figure 1 shows the responses distribution for both parts of the questionnaire.

**Figure 2:**
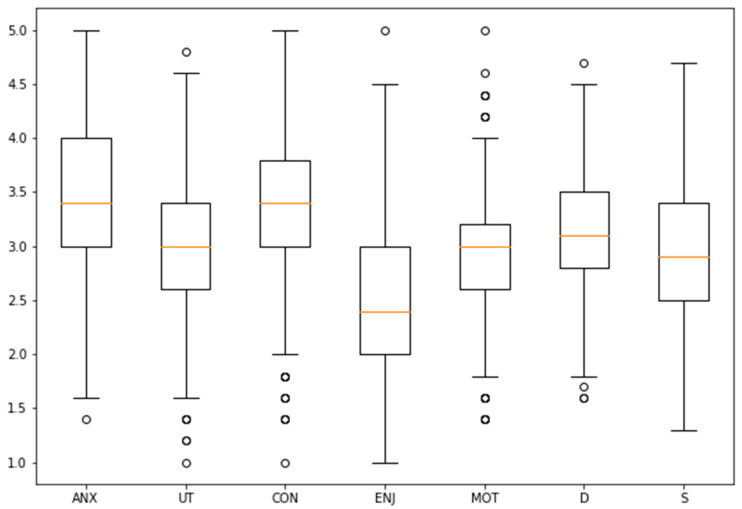
Distribution of students’ dimensions. MSATS: UT = Usefulness (α = 0.81), ANX = Anxiety (reverse coded, α = 0.76); CON = Self-confidence (α = 0.725), ENJ = Enjoyment (α = 0.748); MOT = Motivation (reverse coded, (α = 0.702) and R-SPQ-2F: D = Deep approach (α = 0.715), S = Surface approach (α = 0.723).

We tested the validity of the measure performing a reliability analysis through Cronbach’s Alpha [60]. Figure 2 shows the distribution of the aggregated scales for the 5 dimensions of attitudes towards statistics included in MSATS questionnaire and the two learning approaches in R-SPQ-2F and Cronbach’s alpha is reported next to the dimension label.

For the analysis we used fuzzy sets qualitative comparative analysis fsQCA. We think this approach is appropriate because it seeks for causal combinations of conditions that can lead to the desired outcome [61] and it overcomes some of the problems with regression-based techniques when looking for complex relationships between variables in the model. Furthermore, fsQCA has been widely applied with survey data and samples with a medium/large number of observations in the social sciences [62].

Regression based models are good at evaluating the net impact of one or more independent variables on a dependent variable. The effect and significance of an independent variable varies depending on the other independent variables present in the study [63], in a way, competing to explain more variance than the other independent variables. In fsQCA, the conditions collaborate to obtain the desired outcome [64]. These combinations of causal conditions are sometimes complex and involve several conditions (interactions), which additionally can act in different ways according to the context (asymmetry) [65]. In a regression-based model, context interactions are difficult to interpret and to evaluate separately from the net effects of the other independent variables and interactions, with this difficulty increasing as the number of independent variables involved increases [66]. Additionally, the assumption of symmetric relationships between the dependent and independent variables can hide configurations of conditions that have an effect on the desired outcome [67]. FsQCA handles these limitations of a regression-based model [68] by studying all the different paths or combinations of causal conditions that can lead to the desired outcome.

**Figure 3:**
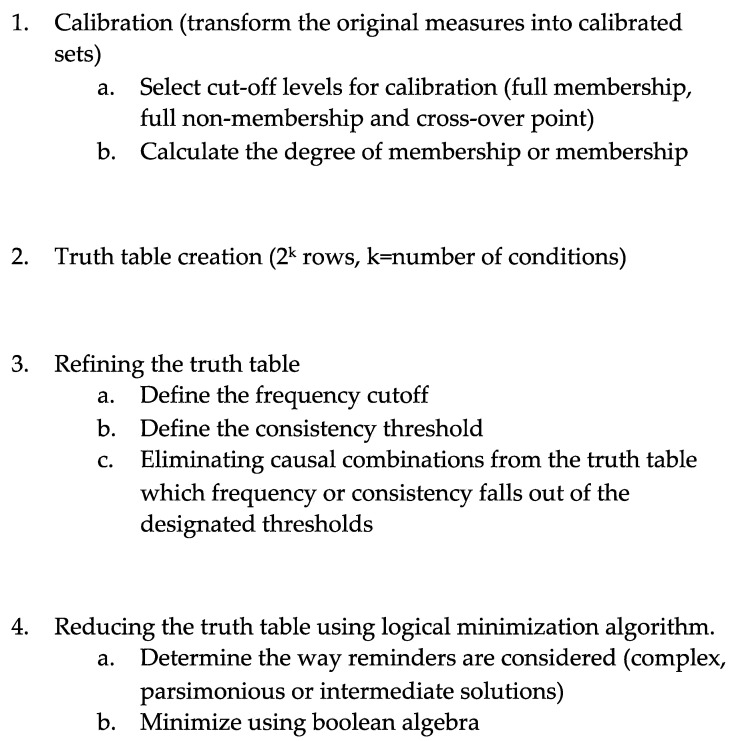
Fuzzy sets comparative qualitative analysis (FsQCA) process.

The fsQCA methodology starts by calibrating the measures (see Figure 3). The calibration is the transformation of the initial scales into fuzzy sets. Fuzzy sets values indicate the membership of a certain individual in a set, and range from 0 for full non-membership to 1 for full membership. Among the different calibration methods, we selected the most common one, the direct method proposed by Ragin [69]. This method consists, first, of the selection of three anchor points: full membership, full non-membership and a cross-over point. The original scores are then converted into fuzzy scores by calculating the degree of membership (see 1) as in Ordanini et al. [70], and considering a full membership to correspond to a membership score of 0.95, the cross-over point to 0.5 and full non-membership to 0.05. The results, ranging from 0 to 1, are new calibrated values representing the extent to which an individual belongs to a specific set.

In our case, as Auzmendi’s [9] instrument and R-SPQ-2F are based on additive scales. Then, we used the average of the suggested items in each of the five dimensions evaluated in Auzmendi’s survey [9] and the ten items for each of the two learning approaches in R-SPQ-2F, to obtain the input values for the calibration process [70,71,72]. Then, taking into account the fact that we were dealing with a relatively large number of cases, we selected the three threshold points following [68,73,74]. These authors suggest using percentiles to determine the anchor points, allowing one to deal with the distinctive distributions that can be found in the data for each of the dimensions under analysis (see Figure 2). We set full membership in the 90% percentile, full non-membership in the 10% percentile and the cross over point in the 50% percentile. Then, we centered the original scores on the cross over point, we transformed them to odds ratios and we calculated the degree of membership using Equation (1) to obtain the desired fuzzy membership score between 0 and 1.

degree of membership = exp(log odds)/(1 + exp(log odds))(1)

Once we had completed the calibration using the fsQCA 3.0 [75,76] software, we produced the truth table (see Figure 3). The truth table includes all the possible combinations of conditions for a desired outcome. It is formed of 2^k^ rows, where k represents the number of conditions in the study that are involved in causing the outcome. In our case, we had four attitudes from the MSATS questionnaire plus two learning approaches from R-SPQ-2F. Therefore, we had 64 possible configurations or combinations of conditions that can lead to the desired outcome. Based on the relatively large number of cases in the sample and their distribution in the truth table, and following Ragin [77], we selected those combinations of conditions with a minimum frequency of 5, and a consistency level of at least 0.8. Thus, only combinations of conditions with at least five observations and a proportion of cases consistent with the desired outcome of 80% or higher were considered for the minimization process (see Figure 3). The logical minimization can be approached in various ways, depending on how logical remainders are handled. Logical remainders are configurations with no cases in the sample data [69,77,78]. Three possible solutions result from the way logical remainders are handled (see Figure 3): the complex, the parsimonious or the intermediate solutions, which, respectively, consider no remainders, all the remainders or just those that are reasonable in the relationship between the conditions and the outcome. In our study, we analyzed the intermediate solution, which has been widely reported [68] as being superior to the others.

## 3. Results

Table 1 summarizes the results of the logical minimization for the intermediate solution that causes the desired outcome. As all the measures were coded positively regarding their effect, we represent, for example, low levels of anxiety towards statistics as ANX and high levels as ~ANX. A ~ sign before a measure therefore indicates the absence (low values) or the opposite of the causal condition or the desired outcome. In Table 1, each row represents a different condition or combination of conditions (configurations), a different path, to the desired outcome. For example, for the model leading to low levels of anxiety (ANX), the first configuration that is considered to be a consistent subset of and sufficient for obtaining lower levels of anxiety is a high level of self-confidence (CON). In other words, self-confidence is a sufficient condition for low levels of anxiety towards statistics. Similarly, the second, third and fourth paths to low levels of anxiety are a combination of high levels of motivation (MOT) and the absence a strongly surface learning approach (~S), combined with any one of a low value for a deep learning approach (~D), utility (~UT) or enjoyment (~ENJ).

In fsQCA we should consider other aspects, such as coverage and consistency, for the analyses. Coverage is equivalent to the explained variance in regression-based models [79], where raw coverage evaluates the proportion of the desired outcome that is explained by that configuration, and unique coverage the proportion that is explained solely by that configuration. Thus, it is a measure of the importance of the configuration in delivering the desired outcome. In our case, the first solution (CON) clearly emerges as the most important, covering 77.4% of the cases with low levels of anxiety, while overall the model explains 83.8% of the cases. In other words, the model captures 83.8% of the students showing low values of anxiety and 77.4% of students with these low values of anxiety showed low levels of self-confidence.

Additionally, we can consider the solution’s consistency. Consistency is equivalent to statistical significance in a regression model [78]. Taking into account the fact that Ragin [68] suggested a cut-off of 0.75 for sufficient consistency, we can say that configurations 2 (MOT • ~S • ~D) and 4 (MOT • ~S • ~UT) are not consistent enough to assume that they are sufficient for achieving low levels of anxiety.

In relation to the objective of the research, we can also evaluate the impact of the learning approach in the reduction of the anxiety. We can see that low levels of anxiety are, in most cases, independent of the learning approach, except in a small number of cases relative to the configuration 3. Indeed, only 28.5% of the cases showing low levels of anxiety also showed low values of a surface approach to learning in combination with a high motivation and low levels of enjoyment and, an insignificant proportion were uniquely represented by this configuration.

Regarding high levels of anxiety (~ANX), the results also reveal that self-confidence is a major driver. The absence of self-confidence (~CON) is, by itself, a sufficient condition for high levels of anxiety towards statistics. This path explains 78.8% of the cases of students showing low levels of anxiety. In other words, almost 79% of the students showing high levels of anxiety also showed absence or low levels of self-confidence. Additionally, students showing a combination of negative attitudes towards statistics, such as, low motivation, low enjoyment and low perceived usefulness, in combination with a low deep and a low surface approach (~UT • ~ENJ • ~MOT • ~D • ~S), also showed consistently (83.3% of the cases) high levels of anxiety. However, this configuration, once again, is rather residual if we look at the solution’s unique and raw coverage; it is only present in 20.4 percent of the cases with high anxiety.

Finally, we performed a necessity analysis. Necessary conditions are conditions that are present in all of the configurations that lead to the outcome. We can consider a condition as necessary if the consistency is 0.9 or higher [77]. Despite the importance of the presence or absence of self-confidence, the necessity analysis discloses that no condition is necessary for reaching, respectively, low or high levels of anxiety.

These results are in line with the results in [29] that highlight the core role of self-confidence in the achievement of lower levels of anxiety.

## 4. Discussion and Conclusions

Educators managing activities in undergraduate courses that involve statistical concepts often see their students struggling and performing poorly in the related tasks. The cause of this situation has been a matter of extensive research. Many studies have focused on evaluating the attitudes of undergraduate students towards statistics, and have found that these are certainly and heavily affected by the students’ previous training and experiences in the area [8]. These experiences have built the students’ self-beliefs about statistics and have conditioned their approach to the related activities. In other words, a student’s statistical background allows them to evaluate how well they can perform the task, defining their expectations. Negative experiences in the area are then perceived by students as a threat to their successful performance of a statistical task [6,22,80] affecting their learning approach, their attitude and their performance.

In this context, the courses that represent the students’ experiences prior to their undergraduate courses are crucial, first, to provide an understanding of the attitude or combination of attitudes that leads to low and high levels of anxiety, and second, to allow interventions and the introduction of corrective measures in the early stages of students’ contact with statistics, with the aim of improving the situation. However, the level of anxiety has also been related to how students learn, which is influenced by their perception of the learning environment, their personal characteristics and their motivation [36]. Thus, the purpose of this work was to study the interrelation between anxiety, attitudes and the learning strategy. More precisely, we looked for combinations of attitudes and learning approaches that result in high or low levels of anxiety. Our results revealed that self-confidence was the major aspect driving anxiety. High levels of self-confidence led to low levels of anxiety and vice versa. Looking for a chain of events, a negative previous experience predisposes a student to have a negative attitude towards the activity. Negative experiences decrease students’ self-confidence, resulting in lower expectations [12]. Students calculate the value of a task on the basis of three aspects: its importance for the course, their expected enjoyment and its utility [12]. A reduction of their expectations and their perception of the value of the task also decreases their motivation [12]. All these negative feelings provoke an increase in the emotions of fear and apprehension towards statistics, which is anxiety, and, eventually, raises barriers to effective learning [6] and successful performance [3,8].

The results of our study are useful to uncover the key role of self-confidence in creating appropriate feelings towards statistics, and therefore, to lead class activities to increase it. As self-confidence is built on students’ previous experiences, we might want to design activities in the first courses that aim to increase students’ self-confidence in dealing with statistics. We might want students to learn at their own speed and level, to understand the fundamentals better and use a more inductive approach, and to seek motivation through activities that can be easily identified with their interests. In this line, we suggest having activities at different levels to fit students’ progression in the subject, using a variety of active methodologies and “hands on” activities, and framing the activities in a context that matters to the student (for example, statistics applied to a sport or the pandemic).

The limitations of our study are related to its scope. We focused on a group of three secondary schools in a specific region. In Spain, the education system is controlled by the regional government, and the contents and concepts related to statistics can vary from one region to another or from a region to other countries. By contrast, a study in three different suburban towns in the same region ensures a homogeneous group of students, in terms of their previous background in the area. Another limitation of the study is related to the numerous external and internal aspects that can affect student behavior. For example, the teacher’s capability, the class environment, the student’s own capabilities, and the teaching methodology are factors that we did not consider but that can have a moderating or direct impact on the relationships in this study. Finally, we carried out the study at a single time point, what prevents us from evaluating the evolution of these variables over time.

Future research might consider extending the analysis to university undergraduate and master students from similar backgrounds to the students in the study, to evaluate the evolution and the relative impact of the anxiety drivers and their performance levels in statistics. We also might consider introducing potential moderating variables that intervene in this relation such as the prevailing teaching methodology or the student perception of their math teacher.

## Figures and Tables

**Table 1:** Configurations for achieving low levels of anxiety (ANX) and high levels of anxiety (~ANX).

Model: ANX = f(Usefulness = UT, Self-confidence = CON, Enjoyment = ENJ, Motivation = MOT, Deep approach = D, Surface approach = S)			
Algorithm: Quine-McCluskey			
Frequency cutoff: 5			
Consistency cutoff: 0.816			
	Raw coverage	Unique coverage	Consistency
Conf.1: CON	0.774	0.459	0.769
Conf.2: MOT • ~S • ~D	0.308	0.009	0.742
Conf.3: MOT • ~S • ~ENJ	0.285	0.002	0.795
Conf.4: MOT • ~S • ~UT	0.301	0.001	0.703
Model: ANX = CON + (MOT • ~S • ~D) + (MOT • ~S • ~ENJ) + (MOT • ~S • ~UT)			
Solution coverage: 0.838			
Solution consistency: 0.725			
Model: ~ANX = f(UT, CON, ENJ, MOT, D, S)			
Algorithm: Quine-McCluskey			
Frequency cutoff: 5			
Consistency cutoff: 0.825			
	Raw coverage	Unique coverage	Consistency
Conf. 5: ~CON	0.788	0.596	0.792
Conf. 6: ~UT • ~ENJ • ~MOT • ~D • ~S	0.204	0.011	0.833
Model: ~ANX = ~CON + (~UT • ~ENJ • ~MOT • ~D • ~S)			
Solution coverage: 0.799			
Solution consistency: 0.779			

Note: Three common operations on fuzzy sets are set negation (logical NOT “~”), set intersection (logical AND “+”) and set union (logical OR “•”).

## Appendix A

The survey comprised the following questions

Age:		Gender:	M/F	Grade:	10th	11th	12th
Average in mathematics:				Overall average grade:			

Part 1 (MSATS)

1. I consider statistics as a very necessary subject in my studies.
2. I’m pretty bad at statistics.
3. Studying or working with statistics doesn’t scare me at all.
4. Using statistics is fun.
5. Statistics is too theoretical for me to be of any use.
6. I want to have a deeper understanding of Statistics.
7. Statistics is one of the subjects that I fear the most.
8. I have confidence in myself when I face a statistical problem.
9. I enjoy talking to others about statistics.
10. Statistics can be useful for those who decide to pursue a science career, but not for other students.
11. Having a good knowledge of statistics will increase my job possibilities.
12. When I face a statistical problem I feel unable to think clearly.
13. I am calm when I face a problem of statistics.
14. Statistics is nice and challenging for me.
15. I hope to have little use of statistics in my professional life.
16. I consider that there are other matters more important than statistics for my future profession.
17. Working with statistics makes me feel nervous.
18. I don’t get upset when I have to work on statistics problems.
19. I would like to have an occupation in which I have to use statistics.
20. It gives me great satisfaction to solve statistical problems.
21. For my professional future, statistics is one of the most important subjects I have to study.
22. Statistics makes me feel uncomfortable and nervous.
23. If I put my mind to it, I think I can master statistics.
24. If I had the opportunity, I would enroll in more statistics courses than are required.
25. The things taught in statistics classes are very uninteresting

Part 2 (R-SPQ-2F)

1. I find that at times studying gives me a feeling of deep personal satisfaction.
2. I find that I have to do enough work on a topic so that I can form my own conclusions before I am satisfied.
3. My aim is to pass the course while doing as little work as possible.
4. I only study seriously what’s given out in class or in the course outlines.
5. I feel that virtually any topic can be highly interesting once I get into it.
6. I find most new topics interesting and often spend extra time trying to obtain more information about them.
7. I do not find my course very interesting so I keep my work to the minimum.
8. I learn some things by rote, going over and over them until I know them by heart even if I do not understand them.
9. I find that studying academic topics can at times be as exciting as a good novel or movie.
10. I test myself on important topics until I understand them completely.
11. I find I can get by in most assessments by memorizing key sections rather than trying to understand them.
12. I generally restrict my study to what is specifically set as I think it is unnecessary to do anything extra.
13. I work hard at my studies because I find the material interesting.
14. I spend a lot of my free time finding out more about interesting topics, which have been discussed in different classes.
15. I find it is unhelpful to study topics in depth. It confuses and wastes time, when all you need is a passing acquaintance with topics.
16. I believe that lecturers shouldn’t expect students to spend significant amounts of time studying material everyone knows won’t be examined.
17. I come to most classes with questions in mind that I want answering.
18. I make a point of looking at most of the suggested readings that go with the lectures.
19. I see no point in learning material, which is not likely to be in the examination.
20. I find the best way to pass examinations is to try to remember answers to likely questions.

## References

[1] Cashin S.E., Elmore P.B. (2005). The survey of attitudes toward statistics scale: A construct validity study. Educ. Psychol. Meas..

[2] Garfield J., Ben-Zvi D. (2007). How students learn statistics revisited: A current review of research on teaching and learning statistics. Int. Stat. Rev..

[3] Gal I., Ginsburg L., Schau C. (1997). Monitoring attitudes and beliefs in statistics education. Assess. Chall. Stat. Educ..

[4] Biens B. (1985). Teaching the Relevance of Statistics through Consumer-Oriented Research. Teach. Psychol..

[5] Estrada A. (2003). Analisis De Las Actitudes Y Conocimientos [Analysis of Attitudes and Knowledge]. Doctoral Thesis.

[6] Gal I., Ginsburg L. (1994). The Role of Beliefs and Attitudes in Learning Statistics: Towards an Assessment Framework. J. Stat. Educ..

[7] Chiesi F., Primi C. (2009). Assessing statistics attitudes among college students: Psychometric properties of the Italian version of the Survey of Attitudes toward Statistics (SATS). Learn. Individ. Differ..

[8] Carmona Márquez J. (2004). Una revisión de las evidencias de fiabilidad y validez de los cuestionarios de actitudes y ansiedad hacia la estadística [A review of the evidence of reliability and validity of the attitudes and anxiety questionnaires towards statistics]. Stat. Educ. Res. J..

[9] Auzmendi Escribano E. (1992). Las Actitudes Hacia la matemática-Estadística en las Enseñanzas Media y Universitaria [Attitudes towards Statistical Mathematics in Secondary and University Education].

[10] Roberts D.M., Bilderback E.W. (1980). Reliability and Validity of a Statistics Attitude Survey. Educ. Psychol. Meas..

[11] Wise S.L. (1985). The development and validation of a scale measuring attitudes toward statistics. Educ. Psychol. Meas..

[12] Schau C., Stevens J., Dauphinee T.L., Vecchio A. (1995). Del The development and validation of the survey of antitudes toward statistics. Educ. Psychol. Meas..

[13] Bayot-Mestre A., Mondéjar-Jiménez J., Mondéjar-Jiménez J.A., Monsalve-Serrano F., Vargas-Vargas M., Misztal M., Trawiński M. (2005). The difficulties of learning concepts in the social sciences. Studies in Teacher Education: Psichopedagogy.

[14] Eccles J., Spence J.T. (1983). Expectancies, values and academic behaviors. Achievement and Achievement Motives: Psychological and Sociological Approaches.

[15] Bandura A. (1999). Self-Efficacy: The Exercise of Control.

[16] Blanco Blanco Á. (2008). Una revisión crítica de la investigación sobre las actitudes de los estudiantes universitarios hacia la Estadística [A critical review of the research on the attitudes of university students towards Statistics]. Rev. Complut. Educ..

[17] Sorge C., Schau C. Impact of engineering students’ attitudes on achievement in statistics: A structural model. Proceedings of the Annual Meeting of the American Educational Research Association.

[18] Wisenbaker J.M., Scott J.S., Nasser F. Structural equation models relating attitudes about and achievement in introductory statistics courses: A comparison of results from the US and Israel. Proceedings of the 9th International Congress on Mathematics Education.

[19] Baloǧlu M. (2003). Individual differences in statistics anxiety among college students. Pers. Individ. Dif..

[20] Benson J. (1989). Structural Components of Statistical Test Anxiety in Adults. J. Exp. Educ..

[21] Carmona J., Martínez R.J., Sánchez M. (2005). Mathematical Background and Attitudes toward Statistics in a Sample of Spanish College Students. Psychol. Rep..

[22] Chiesi F., Primi C. (2010). Cognitive and non-cognitive factors related to students’ statistics achievement. Stat. Educ. Res. J..

[23] Macher D., Paechter M., Papousek I., Ruggeri K. (2012). Statistics anxiety, trait anxiety, learning behavior, and academic performance. Eur. J. Psychol. Educ..

[24] Musch J., Broder A. (1999). Test anxiety versus academic skills: A comparison of two alternative models for predicting performance in a statistics exam. Br. J. Educ. Psychol..

[25] Onwuegbuzie A.J., Seaman M.A. (1995). The Effect of Time Constraints and Statistics Test Anxiety on Test Performance in a Statistics Course. J. Exp. Educ..

[26] Tremblay P.F., Gardner R.C., Heipel G. (2000). A model of the relationships among measures of affect, aptitude and performance in introductory statistics. Can. J. Behav. Sci..

[27] Zeidner M. (1991). Statistics and mathematics anxiety in social science students: Some interesting parallels. Br. J. Educ. Psychol..

[28] Onwuegbuzie A.J., Daley C.E. (1999). Perfectionism and statistics anxiety. Pers. Individ. Dif..

[29] Peiró-Signes Á., Trull Ó., Segarra-Oña M., García-Díaz J.C. (2020). Attitudes towards statistics in secondary education: Findings from fsQCA. Mathematics.

[30] Lalonde R.N., Gardner R.C. (1993). Statistics as a second language? A model for predicting performance in psychology students. Can. J. Behav. Sci. Can. Sci. Comport..

[31] Vigil-Colet A., Lorenzo-Seva U., Condon L. (2008). Development and validation of the statistical anxiety scale. Psicothema.

[32] Budé L., Van De Wiel M.W.J., Imbos T., Candel M.J.J.M., Broers N.J., Berger M.P.F. (2007). Students’ achievements in a statistics course in relation to motivational aspects and study behaviour. Stat. Educ. Res. J..

[33] Roberts D.M., Saxe J.E. (1982). Validity of a Statistics Attitude Survey: A Follow-Up Study. Educ. Psychol. Meas..

[34] Gil Flores J. (1999). Actitudes hacia la estadística. Incidencia de las variables sexo y formación previa [Attitudes towards statistics. Incidence of the variables sex and previous training]. Rev. Española Pedagog..

[35] Schram C.M. (1996). A Meta-Analysis of Gender Differences in Applied Statistics Achievement. J. Educ. Behav. Stat..

[36] Biggs J. (1993). What do inventories of students’ learning processes really measure? A theoretical review and clarification. Br. J. Educ. Psychol..

[37] Clute P.S. (1984). Mathematics Anxiety, Instructional Method, and Achievement in a Survey Course in College Mathematics. J. Res. Math. Educ..

[38] Pulido J.E. (2009). Enseñanza de la estadística a partir de la actitud del alumno. Laurus.

[39] Aparicio A., Bazán J., Martínez G. (2006). Actitud y rendimiento en Estadística en profesores peruanos [Attitude and performance in Statistics in Peruvian teachers]. Proceedings of the Acta Latinoamericana de Matemática Educativa.

[40] Mondéjar Jiménez J., Vargas Vargas M., Mondéjar Jiménez J. (2007). Impacto del uso del e-learning en las actitudes hacia la estadística [Impact of the use of e-learning on attitudes towards statistics]. RELATEC Rev. Latinoam. Tecnol. Educ..

[41] Estrada A. (2011). Instrumentos de medición de actitudes hacia la estadística: La escala EAEE para profesores [Instruments for measuring attitudes towards statistics: The EAEE scale for teachers]. Proceedings of the Investigación en Educación Matemática.

[42] Ramirez C., Schau C., Emmioǧlu E. (2012). The importance of attitudes in statistics education. Stat. Educ. Res. J..

[43] Daley C.E., Onwuegbuzie A.J. The role of multiple intelligences in statistics anxiety. Proceedings of the Anual Meeting of the Mid-South Educational Research Association.

[44] Trull-Domínguez O., Peiró-Signes Á., Segarra-Oña M. (2018). Aprendizaje de herramientas de SPC mediante actividades experienciales y utilizando alubias [Learning SPC tools through experiential activities and using beans]. Proceedings of the Book of Abstracts CIVINEDU 2018: 2nd International Virtual Conference on Educational Research and Innovation.

[45] Peiro-Signes A., Segarra-Ona M.V., Trull-Dominguez O., De-Miguel-Molina B. (2017). Bean bags: An experiential learning activity for quality control. Proceedings of the Proceedings of EDULEARN17 Conference.

[46] Peiró Signes A., Trull Domínguez Ó., del Segarra Oña M.V. (2017). Desarrollo de una actividad experiencial para la enseñanza de estadística [Development of an experiential activity for the teaching of statistics]. Proceedings of the In-Red 2017. III Congreso Nacional de Innovación Educativa y de Docencia en Red.

[47] Trull Dominguez O., Peiro-Signes A., Segarra Oña M., de Miguel Molina M. (2017). Learning design of experiments with catapults. Proceedings of the 10th Annual International Conference of Education, Research and Innovation.

[48] Trull Dominguez O., Peiró-Signes A., Segarra-Oña M., De-Miguel-Molina B. (2017). Enhancing learning of the application of statistical concepts through experiences. Proceedings of the 10th annual International Conference of Education, Research and Innovation.

[49] Johannssen A., Chukhrova N., Schmal F., Stabenow K. (2021). Statistical literacy-Misuse of statistics and its consequences. J. Stat. Data Sci. Educ..

[50] Carnell L.J. (2008). The effect of a student-designed data collection project on attitudes toward statistics. J. Stat. Educ..

[51] Froelich A.G., Stephenson W.R., Duckworth W.M. (2008). Assessment of Materials for Engaging Students in Statistical Discovery. J. Stat. Educ..

[52] Walsh J.J., Ugumba-Agwunobi G. (2002). Individual differences in statistics anxiety: The roles of perfectionism, procrastination and trait anxiety. Pers. Individ. Dif..

[53] Vera O., Díaz C. (2013). Dificultades de estudiantes de psicología en relación al contraste de hipótesis [Difficulties of psychology students in relation to hypothesis testing]. Probab. Condicionada.

[54] Schau C., Millar M., Petocz P. (2012). Research On Attitudes Towards Statistics. Stat. Educ. Res. J..

[55] Gómez Chacón I.M. (2000). Matemática Emocional [Texto Impreso]: Los Afectos en el Aprendizaje Matemático.

[56] Ribes Giner G., Perelló Marín M.R., Pantoja Díaz O. (2017). Literature of the key variables of the co-creation process in higher education institutions. Tec. Empres..

[57] Müller-Merbach H. (2008). Knowledge management: A program for education and leadership. Knowl. Manag. Res. Pract..

[58] Biggs J., Kember D., Leung D.Y.P. (2001). The revised two-factor Study Process Questionnaire: R-SPQ-2F. Br. J. Educ. Psychol..

[59] Fernández Cézar R., Solano Pinto N., Rizzo K., Gomezescobar Camino A., Iglesias L.M., Espinosa A. (2016). Las actitudes hacia las matemáticas en estudiantes y maestros de educación infantil y primaria: Revisión de la adecuación de una escala para su medida [Attitudes towards mathematics in students and teachers of early childhood and primary education: Review. Rev. Iberoam. Ciencia, Tecnol. Soc. CTS.

[60] Nunnally J.C., Bernstein I.H. (1994). Psychometric Theory.

[61] Fiss P.C. (2011). Building Better Causal Theories: A Fuzzy Set Approach to Typologies in Organization Research. Acad. Manag. J..

[62] Emmenegger P., Schraff D., Walter A. QCA, the truth table analysis and large-N survey data: The benefits of calibration and the importance of robustness tests. Proceedings of the 2nd International QCA Expert Workshop.

[63] Hotchkiss E., Smith D.C., Strömberg P. Private equity and the resolution of financial distress. Proceedings of the AFA 2012 Chicago Meetings Paper.

[64] Fiss P.C. (2007). A set-theoretic approach to organizational configurations. Acad. Manag. Rev..

[65] Rihoux B., Ragin C.C. (2008). Configurational Comparative Methods: Qualitative Comparative Analysis (QCA) and Related Techniques.

[66] Braumoeller B.F. (2004). Hypothesis Testing and Multiplicative Interaction Terms. Int. Organ..

[67] Rihoux B. (2006). Qualitative Comparative Analysis (QCA) and Related Systematic Comparative Methods: Recent Advances and Remaining Challenges for Social Science Research. Int. Sociol..

[68] Woodside A.G. (2013). Moving beyond multiple regression analysis to algorithms: Calling for adoption of a paradigm shift from symmetric to asymmetric thinking in data analysis and crafting theory. J. Bus. Res..

[69] Ragin C.C. (2000). Fuzzy-Set Social Science.

[70] Ordanini A., Parasuraman A., Rubera G. (2013). When the Recipe Is More Important Than the Ingredients: A Qualitative Comparative Analysis (QCA) of Service Innovation Configurations. J. Serv. Res..

[71] Palacios-Marqués D., Roig-Dobón S., Comeig I. (2017). Background factors to innovation performance: Results of an empirical study using fsQCA methodology. Qual. Quant..

[72] Pappas I.O., Kourouthanassis P.E., Giannakos M.N., Chrissikopoulos V. (2016). Explaining online shopping behavior with fsQCA: The role of cognitive and affective perceptions. J. Bus. Res..

[73] Dul J. (2016). Identifying single necessary conditions with NCA and fsQCA. J. Bus. Res..

[74] Beynon M.J., Jones P., Pickernell D. (2016). Country-based comparison analysis using fsQCA investigating entrepreneurial attitudes and activity. J. Bus. Res..

[75] Ragin C.C., Davey S. (2016). Fuzzy-Set/Qualitative Comparative Analysis 3.0.

[76] Ragin C.C. (2018). User’s Guide to Fuzzy-Set/Qualitative Comparative Analysis 3.0.

[77] Ragin C.C. (2009). Redesigning Social Inquiry: Fuzzy Sets and Beyond.

[78] Schneider C.Q., Wagemann C. (2010). Standards of Good Practice in Qualitative Comparative Analysis (QCA) and Fuzzy-Sets. Comp. Sociol..

[79] Ragin C.C. (2006). Set Relations in Social Research: Evaluating Their Consistency and Coverage. Polit. Anal..

[80] Comas C., Martins J.A., Nascimento M.M., Estrada A. (2017). Estudio de las Actitudes hacia la Estadística en Estudiantes de Psicología [Study of attitudes towards statistics in psychology students]. Bolema.

